# From Inhalation to Neurodegeneration: Air Pollution as a Modifiable Risk Factor for Alzheimer’s Disease

**DOI:** 10.3390/ijms25136928

**Published:** 2024-06-25

**Authors:** Jordi Olloquequi, Roberto Díaz-Peña, Ester Verdaguer, Miren Ettcheto, Carme Auladell, Antoni Camins

**Affiliations:** 1 Department of Biochemistry and Physiology, Physiology Section, Faculty of Pharmacy and Food Science, Universitat de Barcelona, 08028 Barcelona, Spain; 2 Laboratory of Cellular and Molecular Pathology, Instituto de Ciencias Biomédicas, Facultad de Ciencias de la Salud, Universidad Autónoma de Chile, Talca 3460000, Chile; roberto.diaz.pena@sergas.es; 3 Fundación Pública Galega de Medicina Xenómica, SERGAS, Grupo de Medicina Xenomica-USC, Instituto de Investigación Sanitaria de Santiago (IDIS), 15706 Santiago de Compostela, Spain; 4 Department of Cellular Biology, Physiology and Immunology, Faculty of Biology, Universitat de Barcelona, 08028 Barcelona, Spain; everdaguer@ub.edu (E.V.); cauladell@ub.edu (C.A.); 5 Institute of Neuroscience, Universitat de Barcelona, 08028 Barcelona, Spain; mirenettcheto@ub.edu (M.E.); camins@ub.edu (A.C.); 6 Biomedical Research Networking Center in Neurodegenerative Diseases (CIBERNED), 28031 Madrid, Spain; 7 Institut d’Investigació Sanitària Pere Virgili (IISPV), 43204 Reus, Spain; 8 Department of Pharmacology, Toxicology and Therapeutic Chemistry, Faculty of Pharmacy and Food Science, Universitat de Barcelona, 08028 Barcelona, Spain

**Keywords:** particulate matter, neurodegeneration, dementia, Alzheimer’s disease, neuroinflammation, oxidative stress, neurotoxicity

## Abstract

Air pollution, a growing concern for public health, has been linked to various respiratory and cardiovascular diseases. Emerging evidence also suggests a link between exposure to air pollutants and neurodegenerative diseases, particularly Alzheimer’s disease (AD). This review explores the composition and sources of air pollutants, including particulate matter, gases, persistent organic pollutants, and heavy metals. The pathophysiology of AD is briefly discussed, highlighting the role of beta-amyloid plaques, neurofibrillary tangles, and genetic factors. This article also examines how air pollutants reach the brain and exert their detrimental effects, delving into the neurotoxicity of air pollutants. The molecular mechanisms linking air pollution to neurodegeneration are explored in detail, focusing on oxidative stress, neuroinflammation, and protein aggregation. Preclinical studies, including in vitro experiments and animal models, provide evidence for the direct effects of pollutants on neuronal cells, glial cells, and the blood–brain barrier. Epidemiological studies have reported associations between exposure to air pollution and an increased risk of AD and cognitive decline. The growing body of evidence supporting air pollution as a modifiable risk factor for AD underscores the importance of considering environmental factors in the etiology and progression of neurodegenerative diseases, in the face of worsening global air quality.

## 1. Introduction

The industrial revolution, a pivotal era in technological and societal progress, also marked the beginning of significant environmental challenges. It led to the release of substantial air pollutants, adversely affecting human health. The historical context of air pollution’s impact on public health is vividly illustrated by events such as the Great Smog of London in the 1950s. This incident, caused by a combination of industrial pollution and specific weather conditions, led to a severe public health crisis, and highlighted the urgent need for effective air quality management. Today, air pollution, defined as the alteration of indoor or outdoor environmental quality by various agents, remains a critical global public health concern. This issue, deeply intertwined with social, economic, and legislative factors, as well as lifestyle habits, is exacerbated by the rapid pace of urbanization and industrialization [1]. 

The World Health Organization (WHO)’s data reveals a concerning reality: almost the entire global population (99%) breathes air that exceeds WHO guideline limits, containing high levels of pollutants [2]. This problem is particularly severe in low- and middle-income countries, where the highest levels of exposure are recorded. 

Common sources of air pollution include household combustion devices, motor vehicles, industrial facilities, and forest fires. These sources emit key pollutants like particulate matter (PM), carbon monoxide (CO), ozone (O_3_), nitrogen dioxide (NO_2_), and sulfur dioxide (SO_2_) which are major contributors to several health issues. As a result, air pollution significantly contributes to morbidity and mortality worldwide, accounting for approximately 7 million deaths annually [3]. According to a study by the OECD, the global financial burden of outdoor air pollution could reach $2.6 trillion annually by 2060 [4]. This figure encompasses expenses related to healthcare, lost workdays, and diminished agricultural productivity. Additionally, the welfare costs due to early mortality are projected to increase to around $25 trillion by 2060. The multifaceted nature of global environmental pollution, therefore, poses an unprecedented challenge in our era, demanding urgent and comprehensive responses to mitigate its impact on public health.

Traditionally, the health risks associated with exposure to air pollution have been predominantly linked to respiratory and cardiovascular diseases [5]. These conditions have long been recognized as the primary health concerns stemming from pollutants in the air we breathe. However, in recent years, a growing body of research has begun to shed light on another concerning link: the potential impact of air pollution on neurodegenerative diseases. 

Neurodegenerative diseases are typically categorized as conditions presenting with a range of symptoms including movement disorders, both voluntary and involuntary, as well as behavioral changes, impaired perception, and often significantly reduced communication abilities [6,7]. Since aging is considered the most significant risk factor for neurodegenerative diseases [8], it is not surprising that, within the next 15–20 years, these conditions are expected to surpass cancer and become the second leading cause of death after cardiovascular diseases [9]. This shift is attributed to the growing number of elderly individuals, particularly in developed regions [7,10]. In this respect, the prevalence of dementia is projected to increase from 57,4 million cases globally in 2019 to 152,8 million cases in 2050 [11]. Alzheimer’s disease (AD) causes from 60% to 80% of dementia cases [12], and it is recognized as one of the most prevalent disabilities globally among individuals over 60 years old. 

Emerging studies are increasingly suggesting that long-term exposure to air pollutants may not only affect our lungs and heart but also our brain health [13]. This new area of research is expanding our understanding of the broader implications of air pollution, indicating a possible connection between environmental factors and the development or progression of neurodegenerative conditions. This evolving perspective underscores the need for further investigation into how air pollution contributes to neurological health risks.

## 2. Air Pollution: Composition and Sources

In the context of exploring the intricate relationship between air pollution and neurodegeneration, it is imperative to revisit the types of air pollutants and their respective sources. While comprehensive reviews on this subject exist [1,14,15], we aim to provide a clear framework for subsequent discussions on their roles in AD and to highlight the need of effective strategies for mitigating environmental risk factors associated with air pollution.

Various particles and chemicals can originate from both indoor and outdoor environments, contributing to the air composition. Indoor air pollution (IAP) refers to the accumulation of pollutants released from indoor activities and products in poorly ventilated indoor environments like homes, offices, schools, and other buildings [16]. IAP significantly impacts human health and well-being, a concern highlighted by the WHO, which reports that IAP contributes to an estimated 3.8 million deaths annually [17]. This issue is particularly pronounced in developing and underdeveloped countries, largely due to emissions from the burning of solid fuels such as coal and biomass (including wood, crop residues, and animal dung) [18]. However, IAP is generated by indoor activities prevalent across all regions, such as cooking, smoking, and the use of electronic devices and consumer products, along with emissions from building materials. Notably, this is of utmost importance given that most people spend around 90% of their time indoors, primarily at home or in the workplace [19].

Outdoor or ambient air pollution (OAP) comprises pollutants emitted from natural sources like volcanic eruptions or forest fires, as well as anthropogenic or human-caused sources like vehicles, industries, power generation, waste burning, etc., present in the external environment [20].

In any case, air pollutants can be broadly divided into four categories based on their chemical composition and properties—gaseous pollutants, persistent organic pollutants, heavy metals, and particulate matter (Table 1) [20,21].

### 2.1. Gaseous Pollutants

Gaseous pollutants comprise both inorganic and organic gases emitted into air from natural processes like volcanic eruptions as well as anthropogenic activities involving fuel combustion, chemical processes, and waste degradation, among others. Major gaseous pollutants along with their prominent sources are discussed below.

Carbon monoxide (CO) is a colorless, odorless gas produced primarily from the incomplete combustion of carbon-containing materials. It is also formed through the photochemical conversion of atmospheric methane (CH_4_) and other hydrocarbons. Anthropogenic sources, particularly road transport, have a substantial influence on CO emissions. Indeed, the highest ambient CO concentrations are usually recorded at kerbside and roadside sites, indicating the dominant influence of motor vehicle exhaust emissions [22]. Other sources of CO include industrial processes (e.g., metals processing and chemical manufacturing), solvent use, storage and transport, public power, waste disposal, and recycling [23]. Additional notable sources include the burning of carbon-rich solid fuels like wood, lignin, and biomass for residential cooking and heating in poorly ventilated spaces causing indoor emissions. CO levels in the outside air are typically highest during colder months when inversion conditions are more frequent, trapping air pollutants near the ground beneath a layer of warm air.

The bulk of sulfur oxides (SOx) present in the atmosphere originate from human-generated sources, particularly from coal-burning power stations. Sulfur dioxide (SO_2_) and sulfur trioxide (SO_3_) comprise the sulfur oxide gases mainly produced during combustion of sulfur-containing fuels and wastes including coal, petroleum oils, and metal ores [24]. In the United States, for instance, 65% of SO_2_ releases stem from electrical utilities, with coal-fired facilities making up more than 90% of that portion, whereas, in the European Union, the electricity sector accounts for over 70% of such emissions [25].

Nitrogen oxides, particularly nitric oxide (NO) and nitrogen dioxide (NO_2_), collectively referred to as NOx, play a crucial role in atmospheric chemical processes that determine air quality. These pollutants are of significant concern due to their far-reaching effects, since they participate in reactions that generate secondary pollutants, most notably ozone (O_3_), which is a major component of photochemical smog [26]. NOx are introduced into the troposphere from various biogenic and anthropogenic sources. The primary sources of NOx emissions include industrial processes, vehicular traffic, biomass burning, microbiological emissions from soil, exchange with the stratosphere, lightning, and air traffic. Although current production estimates for some of these sources carry significant uncertainty, it is generally accepted that the majority of total NOx emissions are anthropogenic in origin [27].

Volatile organic compounds (VOCs) is a diverse group of compounds including pure hydrocarbons, which contain only carbon and hydrogen atoms, such as alkanes, alkenes, alkynes, and aromatics. Additionally, VOCs encompass species that contain other elements, including oxygen and chlorine, such as aldehydes, ethers, alcohols, ketones, esters, chlorinated alkanes and alkenes, chlorofluorocarbons (CFCs), and hydrofluorocarbons [22]. Methane (CH_4_) is the simplest VOC in terms of its molecular structure. The majority of anthropogenic methane emissions associated with fossil fuels originate from the extraction, distribution, and consumption of coal, oil, and natural gas. Also, a high proportion of CH_4_ emissions come from ruminant livestock, rice cultivation, and activities related to waste management and disposal, particularly landfill sites [28]. Motor vehicles are the primary anthropogenic source of non-methane volatile organic compounds on a global scale.

Ground level ozone (O_3_) appears as a secondary gaseous pollutant upon complex photochemical reactions between NOx, volatile organics, heat, and sunlight [29]. Vehicles, biomass combustion, fossil fuel power plants, chemical plants, and solvent uses that release ozone precursors play a key role in its formation.

### 2.2. Persistent Organic Pollutants

Persistent organic pollutants (POPs) comprise complex chemical mixtures of organic substances usually containing chlorine, bromine, or fluorine. They are characterized by persistence in environmental matrices, bioaccumulation potential, and toxicity. Persistent POPs encompass a variety of chemical compounds, including polychlorinated dibenzo-p-dioxins (PCDDs), polychlorinated dibenzofurans (PCDFs), and hexachlorobenzene (HCB), as well as organophosphates, dichlorodiphenyldichloroethylene (DDE), and bisphenol A [30]. The semi-volatile nature of POPs allows them to evaporate from soil, dust, and water, to exist as vapor in the air, or to attach to atmospheric particulate matter [31].

Numerous POPs gained widespread use during the surge in industrial production following World War II, a period marked by the introduction of thousands of synthetic chemicals into commercial applications. These substances found significant utility in controlling pests and diseases, enhancing crop production, and facilitating various industrial processes.

### 2.3. Heavy Metals

Heavy metals linked with air pollution comprise trace elements such as lead (Pb), mercury (Hg), cadmium (Cd), chromium (Cr), nickel (Ni), zinc (Zn), manganese (Mn), arsenic (As) and their inorganic salt compounds [32]. The main human-made sources of heavy metals in the atmosphere can be categorized into point sources and diffuse sources. Point sources are specific, identifiable locations from which metals are emitted, such as mines, foundries, smelters, and coal-burning power plants. These industrial activities often involve the extraction, processing, or combustion of materials containing metals, which can lead to their release into the air.

On the other hand, diffuse sources are more widespread and less easily traceable. These include combustion byproducts from various sources, such as industrial processes, residential heating, and waste incineration. Vehicle emissions are another significant diffuse source of metals, as the combustion of fuel and the wear and tear of vehicle parts, such as brakes and tires, can release metal particles into the atmosphere [33].

### 2.4. Particulate Matter

Atmospheric particulate matter (PM) constitutes both solid particles and liquid droplets suspended in air. It primarily consists of carbonaceous particles that have various organic chemicals and reactive metals adsorbed onto their surfaces. The key constituents of PM include sulfates, nitrates, endotoxins, polycyclic aromatic hydrocarbons (PAHs), and heavy metals such as iron, nickel, copper, zinc, and vanadium [34]. Based on aerodynamic diameter, major PM pollutants identified are [35]: PM10 (coarse particles sized ≤10 microns), PM2.5 (fine particles measuring ≤ 2.5 microns) and ultrafine PM (UFPs, submicron particulates < 0.1 micron diameter).

The main sources of airborne PM are quite diverse and can be broadly categorized into primary and secondary origins. Primary sources involve direct emission of particles, with key anthropogenic contributors being road traffic exhaust (especially from older diesel vehicles), non-exhaust vehicular emissions (brake wear, tire abrasion, road dust resuspension), industrial processes (combustion, mining, construction), residential solid fuel burning (wood, coal), and agricultural activities (crop residue burning, soil tillage) [36]. Secondary particles, on the other hand, are formed in the atmosphere via complex chemical reactions, with major precursors being sulfur dioxide, nitrogen oxides, ammonia, and volatile organic compounds [36].

## 3. Neurotoxicity of Air Pollutants: Tracing the Path from Inhalation to the Brain

The mechanism by which inhaled ambient particles lead to systemic effects remains elusive, although it is accepted that air pollutants can impact various organs and tissues beyond the lungs, including the central nervous system (CNS) [37].

Initial research examining the link between air pollution and neurodegenerative diseases focused on feral dogs residing in highly polluted urban settings. These dogs displayed increased oxidative damage and early development of diffuse amyloid plaques, along with a notable rise in DNA damage (specifically apurinic/apyrimidinic sites) across various brain regions including the olfactory bulbs, frontal cortex, and hippocampus [38,39]. Additionally, these canines, when subjected to elevated levels of urban pollutants, exhibited tissue degradation and a deposition of metals like nickel and vanadium within the brain, predominantly following a nasal route (olfactory mucosa to olfactory bulb to frontal cortex) [38]. This pattern not only suggests the nasal pathway as a primary route of entry but also parallels early olfactory impairments observed in AD [40]. These findings initially established the relationship between air pollution exposure and the hastening of pathological features common to neurodegenerative diseases. Even if the translocation rate of air pollutants from their entry site to secondary organs is low, chronic exposure to air pollution can lead to the accumulation of toxic substances in the brain and other secondary target organs over time [41,42].

For instance, the minute size of UFPs allows them to evade the respiratory system’s normal phagocytic defenses, enabling them to traverse both the blood–air barrier and the blood–brain barrier (BBB) and gain entry into the CNS [41,43]. This could be achieved through the translocation of naked particles, uptake by erythrocytes, and ingestion by alveolar macrophages [44,45]. Moreover, air pollutants can directly reach the CNS through sensory nerve endings embedded in airway epithelia or via the olfactory mucosa, and subsequently undergo axonal translocation to ganglionic and CNS structures [46].

In addition to direct translocation, inhaled particles may also exert indirect effects on the CNS and other tissues through the release of systemic inflammatory mediators and oxidative stress products. These substances are produced in the lungs as a result of chronic pollutant-induced damage to the epithelial and endothelial cells and subsequently released into circulation [47].

## 4. Pathophysiology of Alzheimer’s Disease: An Overview

The development of AD is a complex process involving the interplay of various genetic, environmental, and lifestyle factors, although the precise molecular mechanisms underlying the disease remain elusive [48].

AD, like other neurodegenerative disorders, is characterized by the accumulation of toxic protein aggregates, namely beta-amyloid (βA) plaques and neurofibrillary tangles (NFTs) [49,50]. βA plaques are abnormal deposits of the βA peptide that accumulate between neurons, disrupting cell signaling and promoting neuronal damage and death [51]. The accumulation of βA results from an imbalance in its production and clearance, which is derived from the breakdown of the amyloid precursor protein (APP) [52]. The primary neuronal enzyme responsible for the β-cleavage of APP, which leads to the production of amyloidogenic peptides, is BACE1. Consequently, the cleavage of APP by BACE1 is regarded as the first step in the process of βA generation [53]. In turn, NFTs are formed by the aggregation of hyperphosphorylated tau proteins, which normally stabilize microtubules essential for neuronal structure and transport. The formation of tangles disrupts the normal functioning of neurons, leading to their degeneration and death [54].

Genetic variants play a significant role in AD, and they can be classified as risk genes and deterministic genes. Risk genes, such as the apolipoprotein E (APOE) isoform e4, increase the likelihood of developing AD but do not guarantee its onset [55]. Deterministic genes, although rare, have a stronger link to the development of AD. Autosomal dominant mutations in deterministic genes are associated with familial AD (FAD), a form of the disease with a strong genetic component. These rare mutations have been identified in the C99 fragment of the APP, the precursor of βA, or in the protease responsible for its production, known as presenilin (PSEN)/γ-secretase [56]. Recent evidence from animal models of FAD further underscores the complex interplay between genetic factors, amyloid pathology, and neuronal dysfunction across different brain regions [57].

It is hypothesized that the primary neuropathological markers, βA and NFTs, may initiate the neurodegenerative process leading to cortical and hippocampal atrophy. Alongside these markers, multiple pathophysiological processes occur simultaneously, contributing to the progression of AD. These pathological changes include synaptic dysfunction and/or loss, dysregulation of neurotransmitter systems (such as cholinergic and glutamatergic deficits) [58], vascular dysfunction [59], oxidative stress [60], neuroinflammation [61], and metal dysregulation [62]. Understanding these pathophysiological mechanisms is crucial for developing effective prevention approaches, diagnostic tools, and therapeutic strategies to combat AD.

## 5. Molecular Pathways Linking Air Pollution to Neurodegeneration

**Figure 1:**
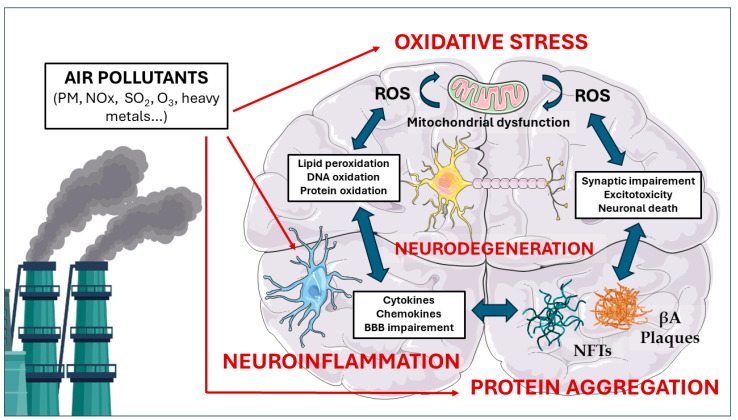
Key molecular pathways linking air pollution to Alzheimer’s disease (AD). Exposure to air pollutants such as particulate matter (PM), nitrogen oxides (NOx), sulfur dioxide (SO_2_), ozone (O_3_), and heavy metals can lead to neurodegeneration and AD pathology through three interconnected mechanisms: oxidative stress, neuroinflammation, and protein aggregation. Air pollutants induce oxidative stress by increasing production of reactive oxygen species (ROS) and decreasing antioxidant defenses in the brain. Oxidative stress causes mitochondrial dysfunction, which further amplifies ROS generation, creating a vicious cycle. Air pollutants also trigger neuroinflammation by activating microglia, which release pro-inflammatory cytokines, chemokines, and more ROS. Activated microglia further compromise the blood–brain barrier (BBB) integrity, which allows the entrance of pro-inflammatory molecules from systemic circulation. Air pollutants are capable of disrupting proteostasis, leading to protein aggregation. Furthermore, the oxidative and inflammatory environment created by air pollutants promotes misfolding and aggregation of β-amyloid (βA) into plaques and tau into neurofibrillary tangles (NFTs), hallmarks of AD pathology. Aβ plaques and NFTs cause synaptic impairment, excitotoxicity, and neuronal death, also promoting oxidative stress and neuroinflammation. These three intertwined pathways initiated by air pollution exposure form a vicious circle, synergistically driving neurodegeneration and the development and exacerbation of AD.

While epidemiological studies have established an association between exposure to air pollution and cognitive impairment, including an increased risk of neurodegeneration, dementia, and AD [63,64,65,66,67], the underlying molecular mechanisms remain to be fully elucidated. To better understand how air pollutants contribute to the development and progression of neurodegenerative disorders, it is crucial to investigate the cellular and molecular pathways that are altered upon exposure. This section will explore the current knowledge on the molecular mechanisms through which air pollution induces neurodegeneration, focusing on the role of oxidative stress, neuroinflammation, and protein aggregation (Figure 1).

Although rare, genetic forms of AD, such as those caused by mutations in the APP, PSEN1, or PSEN2 genes, are characterized by an earlier onset and more aggressive progression compared to sporadic AD [68]. It is plausible that exposure to air pollutants could exacerbate the pathological processes driven by these genetic mutations, potentially through the same mechanisms discussed in our review, such as oxidative stress, neuroinflammation, and protein aggregation. However, to our knowledge, there is currently limited research specifically investigating the interaction between air pollution and genetic forms of AD.

### 5.1. Oxidative Stress

Due to the complex mix of pollutant gases and particles in the air, oxidative stress is often considered a key biological pathway that links the initial molecular triggers to adverse health outcomes. Oxidative stress is defined as a biochemical disequilibrium arising when there is an excess of pro-oxidants, like free radicals or reactive oxygen species (ROS), over the body’s inherent antioxidant defenses, causing oxidative harm. This imbalance is linked to the onset of a wide array of human ailments. Predominantly, ROS act as the chief pro-oxidants. These include radicals like superoxide (O_2_^−^) and hydroxyl (OH^−^) radicals, along with certain non-radical forms originating from O_2_, such as hydrogen peroxide (H_2_O_2_) [69].

The brain is especially susceptible to oxidative stress due to its unique characteristics. It contains high levels of fatty acids, consumes substantial amounts of energy and oxygen, and has relatively weak antioxidant defense systems compared to other organs [70]. These factors make the brain particularly vulnerable to damage caused by ROS. Oxidative stress can manifest in different forms depending on the specific macromolecules targeted by ROS. It can lead to lipid peroxidation, protein oxidation, or DNA oxidation. The accumulation of oxidized lipids, proteins, and DNA by free radicals is thought to contribute to the functional decline observed in aging brains [71,72]. This deterioration is characterized by impairments in cognitive function and motor skills, highlighting the detrimental impact of oxidative stress on neurological health.

Oxidative stress has been proposed to be a key player in the etiology of AD, since it leads to mitochondrial dysfunction, impairments in neuronal bodies and synapses, and contributes to the production of βA [73]. In AD, mitochondrial dysfunction is characterized by impaired mitochondrial complexes responsible for ATP generation, resulting in the production of 4-hidroxinonenal (4-HNE), a marker of lipid peroxidation, and accumulation of βA. Indeed, neurotoxicity in AD is strongly correlated with elevated levels of lipid peroxidation [74]. Furthermore, increased levels of ROS contribute to tau aggregation and hyperphosphorylation [75,76]. Indeed, ROS, amyloid, and tau protein influence the activity of glutamate receptors and uptake, leading to an exacerbated influx of Ca^2+^ in postsynaptic neurons. This increased Ca^2+^ influx further amplifies ROS production, lipid peroxidation, tau phosphorylation, and hyperexcitability, ultimately resulting in the synaptic dysfunction responsible for AD [77,78]. Interestingly, the imbalance of bioactive metals has also been suggested as one of the mechanisms through which oxidative stress influences AD, and inhaled metals, particularly iron (Fe), have been implicated in the pathogenesis of different neurodegenerative diseases, including AD [79,80]. Importantly, excess Fe can lead to ferroptosis, a form of cell death caused by elevated Fe levels, oxidative stress, and lipid peroxidation [81].

Several in vitro and in vivo studies have suggested that air pollutants including fine and ultrafine particles, O_3_, transition metals, SOx, CO, NOx, and PM are linked to oxidative stress in the brain [67,82,83,84,85]. For instance, in a study by Kim et al. [86], the vulnerability of oligodendrocyte precursor cells (OPCs) and mature oligodendrocytes (mOLs) to urban UFP was investigated. The researchers exposed brain cells isolated from neonatal Sprague-Dawley rats to various concentrations of these UFPs and measured survival rates, ROS levels, and total antioxidant capacities. The results showed that OPCs and mOLs were more vulnerable to UFP-induced damage than astrocytes and cortical neurons, with the extent of damage depending on UFP concentration. Furthermore, ROS levels were significantly higher in OPCs and mOLs compared to other brain cell types, while total antioxidant capacity values were significantly lower. These findings suggest that urban UFPs induce oxidative stress in the brain, which particularly impairs adult OPCs and mOLs. This damage may lead to demyelination and reduced remyelination capacity [86].

In another recent study [87], the molecular mechanisms underlying the neurotoxic effects of fine aerosolized PM2.5 were investigated using human SH-SY5Y neuronal cells. The researchers focused on the role of oxidative stress, inflammation, and mitochondrial dysfunction in PM2.5-induced neurotoxicity. The results showed that PM2.5 exposure significantly increased the levels of oxidative stress and led to mitochondrial dysfunction in SH-SY5Y cells. The authors stressed that the impairment of mitochondrial function may further exacerbate the oxidative stress induced by PM2.5 exposure, creating a vicious cycle that ultimately leads to neuronal damage and cell death [87]. Similarly, in another study, homogenized rat brain tissues from the olfactory bulb, cerebral cortex, striatum, hippocampus, and cerebellum were incubated with PM2.5 at various concentrations [88]. The results showed that all PM concentrations caused oxidative damage in the cerebellum and hippocampus, as evidenced by increased lipid peroxidation and decreased catalase (CAT) activity. The striatum and olfactory bulb also exhibited decreased CAT activity, although they did not show higher levels of lipid peroxidation. The authors suggested that the cerebellum and hippocampus are more susceptible to the neurotoxic effects of direct PM exposure, and oxidative stress plays a key role in mediating these effects [88]. It has also been reported that exposure to diesel exhaust particles and urban particles in brain endothelial cells derived from Balb/c mice increases ROS and decreases total antioxidant capacity [89]. Hence, air pollutants can also increase the oxidative stress in the BBB, a matter of importance since alterations in this essential selective barrier are thought to be an early step in AD’s pathology [90].

Regarding in vivo evidence, in a recent study by Bernardi et al., the effects of chronic exposure to ambient air pollution on oxidative stress parameters and the number of neurons and microglial cells in the cortex and striatum of male Wistar rats were investigated [91]. The results showed that the concentration of malondialdehyde, a marker of lipid peroxidation, was significantly higher in the group exposed to air pollution throughout life. Additionally, the activity of the antioxidant enzyme superoxide dismutase was significantly reduced in the cortex of all air pollution-exposed groups [91]. In a study conducted in northern Italy, Milani et al. assessed the impact of UFPs on different brain regions in male BALB/c mice [92]. They examined proteins related to oxidative stress, inflammation, and AD markers in different brain areas such as the hippocampus and cerebellum. The results showed that exposure to UFPs from diesel exhaust resulted in strong oxidative stress and also modulated the levels of APP and BACE1 proteins. In addition, O_3_ exposure has also been shown to cause oxidative stress in the brains of rats, potentially leading to neurodegenerative changes, neuronal death, and memory impairment [93,94,95].

With respect to studies in humans, Guxens et al. [96] used data from the Generation R Study, a population-based birth cohort in Rotterdam, the Netherlands, to investigate the association between exposure to various air pollutants during pregnancy and childhood and brain development in school-age children and pre-adolescents. They reported that exposure to various air pollutants, including NOx, NO_2_, PM10, PM2.5, PAHs, organic carbon, copper, silicon, zinc, and the oxidative potential of PM2.5, was linked to alterations in brain structural morphology, structural connectivity, or functional connectivity [96]. This is a matter of importance, as neurodevelopmental disorders could be linked to AD’s pathophysiology [97].

Overall, these studies consistently highlight that air pollutants can disrupt brain cellular and molecular structures via oxidative mechanisms. These disruptions are able to compromise not only the integrity of neuronal cells but also the overall cognitive functions, underscoring the hazardous oxidative potential of air pollutants in CNS.

### 5.2. Neuroinflammation

Neuroinflammation, an inflammatory response centralized to the brain and spinal cord, is mediated by various cytokines, chemokines, ROS, and other signaling molecules released by microglia (the primary immune surveillance cells in CNS) and astrocytes in response to pathological stimuli [61]. Indeed, activated microglia release pro-inflammatory cytokines such as interleukin-1β (IL-1β), interleukin-6 (IL-6), tumor necrosis factor-α (TNF-α), and chemokines like CXCL-1, CCL2, and CCL5, as well as nitric oxide, prostaglandins, and ROS [98,99]. The net result of ROS-mediated redox signaling in the context of neuroinflammation is typically an enhancement of downstream expression and/or activation of transcription factors that control the expression of more cytokines, chemokines, paracrine molecule-metabolizing enzymes such as cyclooxygenase-2 (COX-II) and arachidonic acid 5-lipoxygenase (LOX), and other ROS-generating enzymes like inducible nitric oxide synthase (iNOS) [100]. Peripheral inflammation also triggers neuroinflammation through the breakdown of the BBB, stimulation of glial cells as part of the systemic immune reaction, and modulation of the autonomic nervous system through the organ–brain axis [101].

The neuroinflammatory response is a critical component in the etiopathogenesis of AD, contributing to the initiation and progression of the disease [102,103]. Hence, it has been shown that AD patients exhibit elevated levels of pro-inflammatory cytokines IL-6 and TNF-α [104,105]. Murine models have further demonstrated that βA deposition increases under inflammatory conditions [106]. Indeed, it has been suggested that elevated cytokine levels in the cerebrospinal fluid (CSF) can diminish the microglia’s ability to absorb βA [107]. Moreover, high levels of prostaglandins have been found to accompany inflammation in AD brains, with PGD2 levels being particularly elevated in the frontal cortex of AD patients compared to controls [108]. Studies have indicated that significant numbers of pro-inflammatory astrocytes are found in the postmortem brain tissues of AD patients, hinting at a malfunction of type A1 reactive astrocytes [109]. Moreover, these reactive A1 astrocytes are known to disrupt the standard functions of the BBB and cerebral blood flow, thereby playing a role in both the onset and advancement of the disease [110].

Interestingly, exposure to heavy metal such as nickel and vanadium, present in higher levels in fine particulate matter, have been consistently linked to neuroinflammation in studies on immortalized microglia cells (BV2) [111]. Other in vitro studies have demonstrated that diesel exhaust particles can activate microglia, and that microglia-derived oxidant species lead to the death of dopaminergic neurons [112]. Diesel exhaust particles have also been shown to cause oxidative stress and increase inflammatory cytokines in brain capillaries in vitro, suggesting that these air pollutants may directly affect the BBB [113]. In this line, a recent study of Seo et al. used a novel “brain-on-a-chip” platform incorporating neurons, glial cells, and brain endothelial cells (bECs) in a neuro-glia-vascular (NGV) model to closely study the interactions under the stress of diesel exhaust particle exposure [114]. The authors reported that granulocyte-macrophage colony-stimulating factor (GM-CSF) secreted by bECs plays a critical role by activating microglia, which in turn overproduce ROS, implicating a cascade of bEC–microglia–neuron interactions culminating in βA accumulation, tau phosphorylation and neuronal death [114]. Another study showed that exposure of primary neuronal cultures to PM2.5 or supernatants from PM2.5-treated macrophages and microglia resulted in decreased neuronal viability and loss of neuronal antigens [115]. The neurotoxicity was found to be mediated by increased extracellular glutamate levels, which were negatively correlated with neuronal viability. They also showed that PM2.5 can impair the tight junctions of endothelial cells, increasing permeability and monocyte transmigration across the endothelial monolayer, suggesting that PM2.5 can disrupt the BBB [115].

In a recent study, Han et al. elegantly investigated the impact of atmospheric PM on demyelinating conditions using a murine experimental autoimmune encephalomyelitis model and primary cultures of microglia, astrocytes, and oligodendrocyte progenitor cells [116]. They found that PM exposure intensified neuroinflammation, damaged myelin, and impaired movement coordination, due to an increase in pro-inflammatory activities of microglia. They also identified the TLR-4/NF-kB signaling pathway as a crucial mediator in this process, orchestrating a network of genes that promote the pathogenicity of microglia when triggered by PM [116]. Other studies in animals have also confirmed the neuroinflammatory potential of air pollutants. As previously explained, dogs living in Mexico City, which is known for its high levels of air pollution, exhibit signs of chronic inflammation, neurodegeneration, and DNA damage in various regions of their brains [39]. Similar findings have been observed in mice exposed to concentrated PM, with increased levels of cytokines and the transcription factor NF-κB in their brains, indicating the presence of neuroinflammation [117]. ApoE^−/−^ mice exposed to UFP also showed elevated levels of NF-κB, along with increased activation of mitogen-activated kinase pathways and glial fibrillary acidic protein (GFAP) [118]. In turn, exposure to traffic-related air pollution in a highway tunnel setting led to heightened levels of pro-inflammatory cytokines, such as IL-1β and IL-6, in the brains of mice [119], while long-term exposure to diesel exhaust in rats resulted in increased concentrations of several pro-inflammatory cytokines in various brain regions, including the striatum [120]. Even short-term exposure to high levels of diesel exhaust in rats induced similar increases in pro-inflammatory cytokines and other enzymes in both the brain and lungs [121].

Regarding the neuroinflammatory potential of other air pollutants, a study of Yao et al. investigated the effects of chronic SO_2_ inhalation on neuronal function in male Wistar rats [122]. The rats were exposed to SO_2_ at concentrations 4–8 times higher than the EPA standard for 90 days. SO_2_ inhalation increased the release of inflammatory cytokines in the brain and impaired spatial learning and memory in the Morris water maze test. The exposed rats also exhibited reduced expression of activity-regulated cytoskeletal associated gene (Arc), glutamate receptor subunits, and memory-related kinases such as PKA, PKC, and CaMKIIα in the hippocampus. These authors suggested that long-term exposure to high levels of SO_2_ can lead to neurobiological changes that are closely linked to behavioral disturbances in spatial learning and memory [122].

O_3_ exposure has also been linked to neuroinflammation in rodents. Hence, Velázquez-Pérez et al. recently reported that low-ozone exposure alters the brain P2X7 receptor, which is involved in inflammation and energy metabolism in rats [94]. Specifically, the results showed changes in P2X7 protein levels, increased phosphorylation of glycogen synthase kinase 3-β (GSK3-β), and alterations in inflammatory cytokines (increased IL-1β and IL-17, decreased IL-10). Additionally, neuronal glycogen accumulation and increased caspase 3 were observed. The findings suggest that repeated exposure to low-ozone doses, as experienced during highly polluted days, induces oxidative stress, leading to P2X7 receptor alterations, inflammatory processes, and cell death, potentially contributing to progressive neurodegeneration, similar to what may occur in AD [94].

Regarding evidence of increased neuroinflammation due to air pollution exposure in humans, Calderón-Garcidueñas et al. investigated autopsy samples of the frontal cortex from control and pollution-exposed children and young adults through RT-PCR and microarray analysis [123]. They assessed gene expression changes in oxidative stress, DNA damage signaling, NFκB signaling, inflammation, and neurodegeneration pathways. Exposed urbanites displayed differential (>2-fold) regulation of 134 genes and upregulated gene network clusters including IL1, NFκB, TNF, IFN, and TLRs [123].

In conclusion, there is growing evidence that exposure to various air pollutants, such as PM, diesel exhaust, sulfur dioxide, and ozone, can trigger neuroinflammation in the brain. These pollutants appear to activate microglia and astrocytes, leading to increased production of pro-inflammatory cytokines, chemokines, and reactive oxygen species.

### 5.3. Protein Aggregation

Air pollutants could contribute to the accumulation and aggregation of abnormal protein fibrils found in AD through different means. As explained above, exposure to air pollutants can lead to an increased production of ROS and pro-inflammatory cytokines in the brain, creating a cellular environment that favors the misfolding and aggregation of proteins [124,125]. Indeed, post-mortem studies of amyloid plaques have provided evidence for the accumulation of redox metals, such as copper, iron, and zinc, suggesting a connection between these metals and βA [126]. In this sense, iron, aluminum, zinc, and copper have been shown to promote βA aggregation [127,128], while redox-active transition metals may promote tau phosphorylation [129]. Moreover, oxidative stress stimulates BACE1 expression, promoting production of pathological levels of βA through a mechanism involving gamma-secretase activity and the JNK/c-jun pathway [130,131]. In turn, activated microglia produce ROS, leading to the heightened activation of genes associated with inflammation [132]. Therefore, neuroinflammation and oxidative stress have the potential to perpetuate each other, especially within the AD context, which could also enhance protein aggregation. Moreover, environmental toxicants, including pesticides, air pollutants, and heavy metals, have been implicated in the disruption of protein homeostasis. These pollutants can cause significant harm to the native conformation of proteins, leading to either a loss of function or the acquisition of toxic properties. Recent evidence indicates that these toxicants can impair the function of molecular chaperones and the proteasomal degradation pathway, two key components of the protein quality control network responsible for maintaining proper protein folding and eliminating misfolded or damaged proteins [133,134].

As explained above, accumulation of βA plaques and tau protein tangles in the brain are critical markers of AD’s pathology. Some studies have provided evidence that exposure to air pollutants can facilitate the development of these toxic aggregates, even in early life. For instance, Haghani et al. investigated the potential link between gestational exposure to air pollution and the increased risk of cognitive aging and AD in later life [135]. They used nano-sized urban particulate matter (nPM) to examine its developmental effects on mice and Caenorhabditis elegans. In C57BL/6J mice, the researchers found that gestational exposure to nPM caused sex-specific gene expression changes in the cerebral cortex of pups at postnatal day 5. These changes were related to DNA damage, oxidative stress, and immune responses, with potential upstream regulators including AD-related genes such as APP, Psen1, and tau. Furthermore, in the nematode model, developmental exposure to nPM in the L1 stage resulted in an accelerated paralysis phenotype in adults, indicating increased βA42 production [135]. These findings provide novel experimental evidence suggesting mechanisms by which developmental exposure to air pollution could increase the risk of AD in later life.

In a study conducted by Calderón-Garcidueñas et al., the association between living in cities with high air pollution and neuroinflammation/neurodegeneration in healthy children and young adults who died suddenly was investigated [136]. They analyzed brain samples from individuals aged around 25 years old, comparing those from low and highly exposed residents. Notably, βA42 immunoreactivity was observed in a significant proportion of subjects younger than 25 years old, particularly in those carrying the APOE 4 allele, while alpha-synuclein, an aggregate related to Parkinson’s disease (PD), was detected in nearly a quarter of this age group. These findings suggest that exposure to air pollution leads to accumulation of βA42 and alpha-synuclein, beginning in childhood. The authors proposed that air pollution should be considered a risk factor for AD and PD, with APOE 4 allele carriers potentially having a higher risk of developing AD if they live in polluted environments.

Several studies conducted in rodents have also highlighted the potential of air pollutants to increase βA aggregation. Hence, mice exposed to an atmosphere enriched with nickel nanoparticles for a duration of 3 h experienced significant increases in brain levels of βA peptides, specifically βA40 and βA42, as well as the ratio of βA42/40. The observed elevations ranged from 72% to 129% compared to control mice [137]. In a recent study of Sahu et al., C57BL/6;C3H wild-type and APP/PS1 mice were exposed to either filtered air or PM2.5 for 6 h per day, 5 days per week, for a period of 3 months [138]. The results showed that PM2.5 exposure significantly increased the βA plaque load in the hippocampus of APP/PS1 mice compared to their filtered air-exposed controls. The levels of PS1 and BACE proteins were also elevated in APP/PS1 mice exposed to PM2.5 [138].

In another study, the effects of co-exposure to ambient PM2.5 and some gaseous pollutants (O_3_, SO_2_, and NO_2_) on the accumulation of βA1-42 in rats were assessed [139]. The researchers also measured the concentrations of metals (Al, Ca, Na, Cr, Mn, Pb, Cd, Ni, Fe, and Cu) in PM2.5 using inductively coupled plasma mass spectrometry and polycyclic aromatic hydrocarbons (PAHs) using gas chromatography–mass spectrometry. Exposure to ambient PM2.5 plus gaseous pollutants significantly increased the concentration of βA1-42 in the hippocampi of rats after 3 months [139]. In this respect, Ku et al. [140] demonstrated that co-exposure to PM2.5 and SO_2_, air pollutants frequently found in coal-burning regions, increased tau phosphorylation in C57BL/6 mice and primary cortical neuron cultures. Notably, these effects were not evident when the pollutants were tested separately at the same doses. Moreover, the study identified a specific microRNA, miR-337-5p, which is similar to a human microRNA that targets tau, as a key player in the combined effect of PM2.5 and SO_2_, contributing to the synergistic neurodegenerative impact [140].

Indeed, it has been proposed that SO_2_ may disrupt the structure of βA fibrils [141]. This disruption occurs through SO_2_’s binding to the peptide backbone and the side chains of charged residues within the βA17-42 peptides. Such interactions are likely to decrease hydrophobic interactions and disturb the electrostatic interactions among charged residues. This destabilization may encourage the proliferation of βA fibrils, thereby potentially playing a role in the progression of AD [141]. In another approach using molecular dynamics, Kaumbekova et al. investigated the effect of UFPs and common atmospheric pollutants on the βA16-21 segment of the βA1-42 peptide [142]. They studied the aggregation kinetics and the formation of beta sheets in the secondary structure of eight βA16-21 peptides in the presence of varying concentrations of NH_4_^+^ and SO_4_^−2^ ions. The study revealed that UFP had an inhibitory effect on the formation of beta sheets in the peptides. However, there appeared to be a synergistic effect between the concentration of (NH_4_)_2_SO_4_ salt and the presence of the UFP on the aggregation kinetics of the peptides [142]. In a subsequent study, the same authors assessed the effect of Benzo[a]Pyrene, a typical PAH, on the oligomerization of βA42 peptides [143]. The simulations revealed that 5.00 mM Benzo[a]Pyrene accelerated the formation of peptide tetramers by 30% and stabilized the C-terminus in βA42 peptides, suggesting a potential exacerbation of AD progression.

In a pivotal experiment, Cacciottolo et al. proposed a mechanistic pathway through which air pollution particulates exacerbate the production of neurotoxic βA peptides, implicating neuronal lipid rafts as critical and novel targets for the oxidative and pro-amyloidogenic impacts of environmental pollutants [144]. They used J20 mice and N2a cells genetically modified to express human APP with the Swedish mutation (hAPPswe)—a familial form of AD—exposed to nano PM. In J20-APPswe mice, a 150-h exposure to nPM led to significant increases in lipid oxidation products, such as 4-HNE, within the lipid raft fractions of the cerebral cortex. This biochemical change facilitated the pro-amyloidogenic processing of APP, thereby increasing βA peptide production. Notably, these alterations were not observed in the cerebellum, highlighting the region-specific vulnerability similar to that seen in AD, where βA deposits are predominantly found in the cerebral cortex and not the cerebellum. Further in vitro investigations with N2a-APPswe cells confirmed these findings, demonstrating dose-dependent oxidative responses to nano PM, including alterations in the lipid raft-associated APP processing that favor βA peptide production [144].

The influence of O_3_ exposure on βA plaque pathology has also been recently examined in the murine model of AD 5xFAD mice by Greve et al. [145]. They found that O_3_ exposure impaired the ability of microglia to associate with and form a protective barrier around βA plaques. This impairment led to an increase in dystrophic neurites and βA plaque load. Interestingly, spatial proteomic profiling analysis revealed that O_3_ exposure caused dysregulation of disease-associated microglia protein expression and increased levels of pathogenic molecules in the peri-plaque microenvironment.

Epidemiological evidence also highlights the potential impact of air pollution on the formation of βA. For example, in the already discussed study by Calderón-Garcidueñas et al. [123], they showed that 51% autopsy brain samples of air-pollution exposed individuals exhibited βA diffuse plaques, compared to 0% in controls. A recent study involving 3,029 participants aged 75 and older, all dementia-free at the start, explored the relationship between long-term exposure to air pollutants and plasma βA levels [146]. The researchers assessed the participants’ exposure to PM2.5, PM10, and NO_2_ at their residential addresses using validated spatiotemporal models covering up to 20 years before plasma collection. The findings revealed that an increase of 3 µg/m^3^ in PM10 exposure over a decade was correlated with a 1.80% increase in βA1-40 levels at baseline. In longitudinal analyses, this association was even more pronounced, showing a 5.20% rise in βA1-40 levels. Similar trends were observed with PM2.5 and NO_2_ exposures in the repeated measures analyses [146]. In another study, Iaccarino et al. examined the association between the likelihood of amyloid positron emission tomography (PET) scan positivity and ambient air quality in individuals with cognitive impairment [147]. The study included 18,178 US participants with mild cognitive impairment (MCI) or dementia who received an amyloid PET scan. Air pollution was estimated at the patient’s residence using predicted PM2.5 and ground-level O_3_ concentrations. The study found that living in areas with higher estimated biennial PM2.5 concentrations was associated with a higher likelihood of amyloid PET scan positivity, after adjusting for various demographic, lifestyle, socioeconomic, and medical factors [147]. Similar results have been recently reported by Duchesne et al., who evaluated plasma βA levels at baseline and at 10 years in 287 cognitively unimpaired participants of the Three-City study [148]. The authors estimated exposure to PM2.5, NO_2_, and black carbon at participants’ residential addresses over the 5 years preceding the baseline visit using land-use regression models. The study found that exposure to the three air pollutants was associated with a decreased βA1-42/βA1-40 ratio at baseline. These results seem to corroborate previous findings on the detrimental effect of air pollution on cognition, as a decreased plasma βA ratio may predict AD.

In conclusion, the evidence presented strongly suggests that exposure to air pollutants can contribute to the accumulation and aggregation of abnormal protein fibrils found in AD through various mechanisms. Both preclinical and epidemiological studies have provided evidence that exposure to air pollutants, such as PM, SO_2_, and O_3_, can facilitate the development of toxic aggregates, even in early life. These findings underscore the potential role of air pollution as a risk factor for AD.

## 6. Conclusions

The evidence from preclinical studies, including in vitro experiments and animal models, provides valuable insights into the molecular mechanisms underlying the neurotoxicity of air pollutants.

Oxidative stress emerges as a key mechanism linking air pollution to neurodegeneration. Exposure to pollutants such as PM, O_3_, and SO_2_ leads to an increased production of ROS and a decrease in antioxidant defenses, creating an imbalance that contributes to neuronal damage and dysfunction. This oxidative stress not only directly affects neurons but also impairs the BBB, facilitating the entry of pollutants and inflammatory mediators into the brain. Neuroinflammation, another critical pathway, is triggered by air pollutants through the activation of microglia and astrocytes. These glial cells release pro-inflammatory cytokines, chemokines, and ROS, perpetuating a cycle of inflammation and oxidative stress. The resulting chronic neuroinflammation contributes to the development and progression of AD. Air pollutants also play a significant role in the accumulation and aggregation of abnormal protein fibrils, such as βA and tau, which are hallmarks of AD pathology. Exposure to pollutants can create a cellular environment that favors the misfolding and aggregation of these proteins, leading to the formation of neurotoxic plaques and tangles. Moreover, air pollutants can disrupt protein homeostasis by impairing the function of molecular chaperones and the proteasomal degradation pathway, further contributing to the accumulation of abnormal proteins.

In conclusion, the growing body of evidence presented in this review article highlights the significant role of air pollution as a modifiable risk factor for AD and other neurodegenerative disorders. The complex mixture of particulate matter, gases, and organic compounds in polluted air, mostly of anthropogenic origin, has been shown to exert detrimental effects on the central nervous system through various molecular pathways. The identification of oxidative stress, neuroinflammation, and protein aggregation as key molecular pathways linking air pollution to neurodegeneration provides valuable insights into potential therapeutic targets and preventive strategies. As the global burden of AD continues to rise, it is crucial to recognize the impact of environmental factors and to develop effective interventions to mitigate the risk posed by air pollution. Future research should focus on further elucidating the complex interactions between air pollutants, genetic susceptibility, and other risk factors to develop targeted approaches for the prevention and management of neurodegenerative diseases in the context of a rapidly changing environment.

## Figures and Tables

**Table 1:** Composition and sources of major air pollutants.

Category	Pollutants	Main Sources
Gaseous Pollutants	Carbon monoxide (CO), sulfur oxides (SOx), nitrogen oxides (NOx), volatile organic compounds (VOCs), ozone (O_3_)	Incomplete combustion of carbon-containing materials, coal-burning power stations, industrial processes, vehicular traffic, biomass burning, photochemical reactions
Persistent Organic Pollutants (POPs)	Polychlorinated dibenzo-p-dioxins (PCDDs), polychlorinated dibenzofurans (PCDFs), hexachlorobenzene (HCB), organophosphates, dichlorodiphenyldichloroethylene (DDE), bisphenol A	Widespread use in controlling pests and diseases, enhancing crop production, and facilitating various industrial processes
Heavy Metals	Lead (Pb), mercury (Hg), cadmium (Cd), chromium (Cr), nickel (Ni), zinc (Zn), manganese (Mn), arsenic (As), and their inorganic salt compounds	Point sources: mines, foundries, smelters, coal-burning power plants; diffuse sources: combustion byproducts from industrial processes, residential heating, waste incineration, vehicle emissions
Particulate Matter (PM)	PM10 (coarse particles, ≤10 μm), PM2.5 (fine particles, ≤2.5 μm), ultrafine PM (UFPs, <0.1 μm); composed of sulfates, nitrates, endotoxins, polycyclic aromatic hydrocarbons (PAHs), heavy metals (iron, nickel, copper, zinc, vanadium)	Primary sources: road traffic exhaust, non-exhaust vehicular emissions (brake wear, tire abrasion, road dust resuspension), industrial processes (combustion, mining, construction), residential solid fuel burning (wood, coal), agricultural activities (crop residue burning, soil tillage); secondary particles: complex chemical reactions in the atmosphere
